# Addressing the maldistribution of health resources in Sichuan Province, China: A county-level analysis

**DOI:** 10.1371/journal.pone.0250526

**Published:** 2021-04-23

**Authors:** Li Ding, Ning Zhang, Ying Mao

**Affiliations:** 1 School of Humanities and Social Science, Xi’an Jiaotong University, Xi’an, Shaanxi, China; 2 Health Commission of Xi’an, Xi’an, Shaanxi, China; 3 School of Public Policy and Administration, Xi’an Jiaotong University, Xi’an, Shaanxi, China; 4 Research Center for the Belt and Road Health Policy and Health Technology Assessment, Xi’an Jiaotong University, Xi’an, Shaanxi, China; 5 Centre for Global Infectious Disease Analysis, Department of Infectious Disease Epidemiology, Imperial College London, London, United Kingdom; Georgia Southern University, UNITED STATES

## Abstract

**Introduction:**

The equity of health resource allocation geographically is a contested topic. Sichuan Province, located in Southwest China, has varied topography, providing us with natural materials to explore the determinants of health resource distribution.

**Materials and methods:**

Spatial panel econometric models were constructed to explore the relationship between health resources and factors such as health care service demand and socioeconomic and demographic perspectives using data from Sichuan Province for eight consecutive years (2010–2017).

**Results:**

Health care service demands were found to be a major driving force behind the distribution of health resources, showing that an increase in health care service demands draws health resources to specific counties and surrounding areas. From a socioeconomic perspective, gross domestic product per capita and the average wage show a positive association with health resources. In addition, the total population and proportion of the urban population have diverse effects in regard to health-related human resources but have the same effects on material and financial health resources.

**Conclusions:**

Our results provide the Chinese government with evidence needed to formulate and promulgate effective policies, especially those aiming to tackle inequity among different regions.

## Introduction

Health equity, according to the definition developed by the World Health Organization (WHO) [1], implies providing everyone regardless of gender, race, religion, sexual orientation, nationality, political affiliation, or other characteristics a fair opportunity to attain their full health potential without barriers [2]. Health equity is typically described in terms of horizontal and vertical equity [3]. The equity of health resource allocation geographically is a contested issue in regard to health equity [4, 5], which is defined as the workforce, facilities, revenue, equipment, and supplies available to produce requisite health care and services among different regions, covering the human, material and financial resources in examined the present study [6].

The regional maldistribution of health resources has been a major challenge for a long time in China and has been observed in other areas worldwide, including the United States [7], Sweden [8], Indonesia [9], Thailand [10], Iran [11], and Malawi [12]. As the largest developing country in the world, China has the world’s largest health resource volume, covering a wide range of geographic areas with unbalanced economic development, thus serving as a typical setting for studying ways to address the maldistribution of health resources. Sichuan Province is located in Southwest China, which has varied topography and includes the Qinghai-Tibet Plateau, Hengduan Mountains, Yunnan-Guizhou Plateau, Qinling Mountains, and Sichuan Basin. The considerable differences between these different regions provide us with a natural setting to explore the determinants of health resource distribution.

Most importantly, what factors affect the regional maldistribution of health resources in China? Existing studies have researched such determinants from microcosmic and macroscopic views or from an individual focus on the behaviors and intentions of health-related human resources and from regional variations in all health resources, respectively. Regarding the allocation of health-related human resources, Liu et al., using the individual perspective, carried out several studies on the distribution of the health workforce and found age, education level, income level, job satisfaction, and work stress to be associated with turnover intentions in Western China [13, 14]. Zhang collected data from 2426 towns in Sichuan Province and used structural equation modeling to examine the relationship between job satisfaction and turnover intentions [15]. Zhu et al., from a macroscopic perspective, analyzed the relationship between health-related human resources and the demand for health care services, health investment, and education capacity based on the theory of supply and demand [16, 17]. Focusing on health-related material resources, Pan et al. analyzed the relationship between bed density and savings per capita, government revenues, urban population size, and area size [18] and found that both savings per capita and government revenue show a strong positive relationship with hospital bed density. Sun found that the distribution of high-technology medical equipment varies regionally and tends to concentrate in metropolises [19]. The author conducted a distribution equity assessment of computed tomography (CT) and magnetic resonance imaging (MRI) scanners [20]. Regarding health-related financial resources, Xie et al. researched the equity of health resource distribution in China and identified that health investment from the government, society and individuals is positively associated with wealth within a region [21].

Researchers have explored the equity of health resources and their determinants in China. However, there has been a lack of analysis related to the exploration of distribution and its determinants in Sichuan Province. To fill this research gap, our study measures the determinants of health resources based on spatial panel econometric models. Health resources include health human, material, and financial resources [22]. In the context of China, health-related human resources include of licensed doctors, registered nurses, registered pharmacists, technologists, interns, and so on. We selected licensed doctors and registered nurses as representative human resources. The bed density of medical institutes was used to reflect health-related material resources [18]. Due to issues of data availability, we include the income of medical institutes rather than health investments from government, society, and individuals as a proxy variable of health-related financial resources. Almost all related investments contribute the incomes of medical institutes. The present work had the following objectives: (1) to test and compare the effects of variables in terms of health care service demand, economic conditions, and population; (2) to showcase and compare the spillover effects of different factors to surrounding units on different health resources; and (3) to reveal and compare the direct and indirect effects of different variables.

## Materials and methods

### Study area

**Fig 1:**
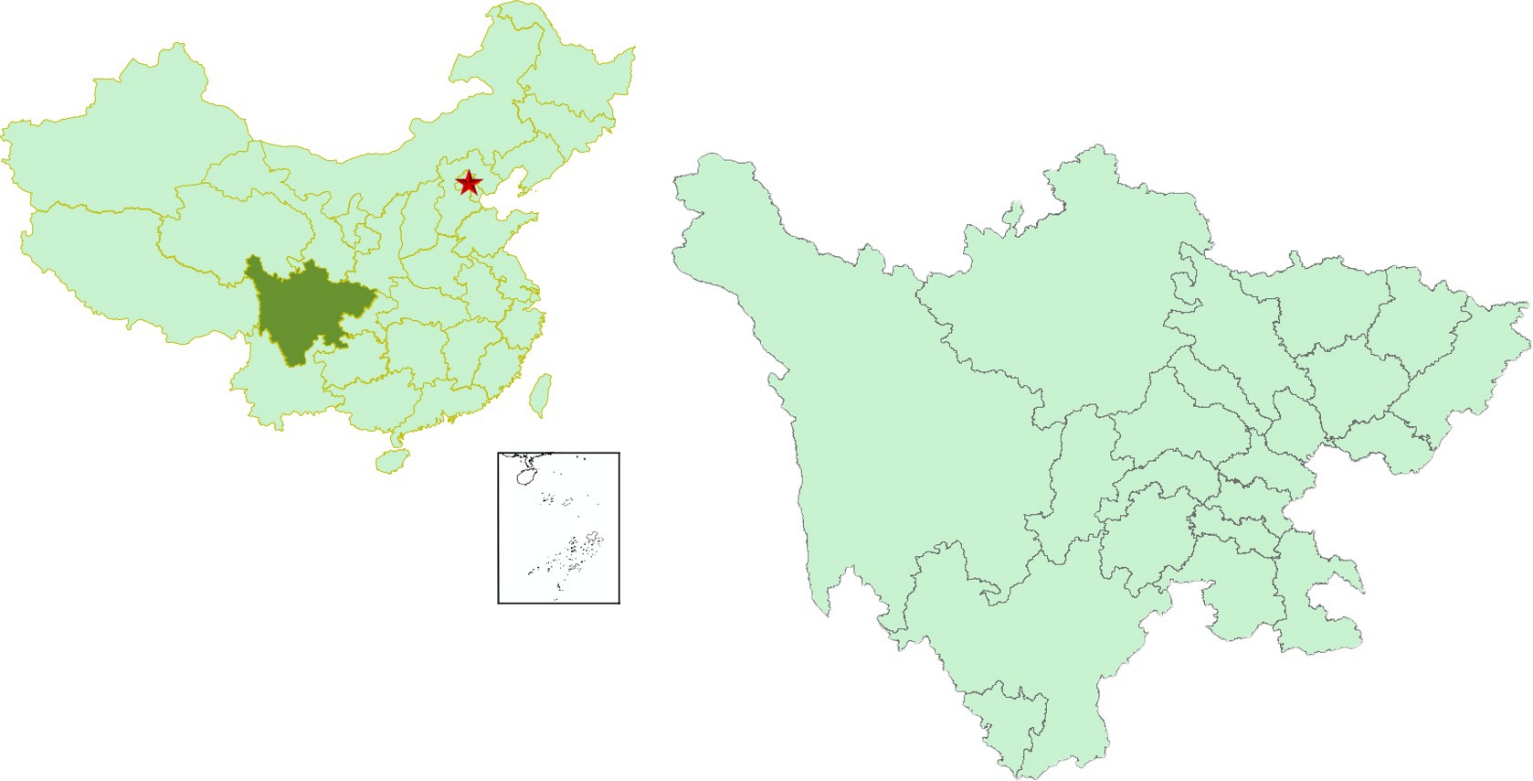
Map of Sichuan. * The source of shape files was a public database, National Nature Resources and Geospatial basic information database of PRC (http://www.geodata.gov.cn/web/geo/index.html). Those shape files were under license without need for permission.

Sichuan is one of 34 provincial administrative regions of China and its capital in Chengdu, which is located in interior Southwest China. Sichuan Province is characterized by considerable topographic variation. The local terrain is of high altitude in the west and low altitude in the east and includes mountains, hills, plain basins, and plateaus. Sichuan Province covers a total area of 486,000 square kilometers and had a population of 83.41 million and a GDP of 4,067.813 billion RMB at the end of 2018. A map of Sichuan is shown in Fig 1 (the map is our own).

### Data source

This study applied county-level data on health resources and socioeconomic characteristics for 2010–2017 for Sichuan. S1 Fig displays the administrative and topographic units of Sichuan Province. Health resource data were obtained from the Sichuan Health Statistics Yearbook (SHSY) and Sichuan Health and Family Planning Statistical Yearbook (SHFPSY) published by the Health Commission of Sichuan Province, while socioeconomic data were obtained from the Sichuan Statistical Yearbook (SSY) published by the Sichuan Bureau of Statistics and the China County Statistical Yearbook (CCSY) published by the National Bureau of Statistics of China. All of these data are publicly available.

County-level administrative divisions in Sichuan changed over the research period. The Enyang and Qianfeng districts were established in 2013. Hence, of the 183 administrative districts present in Sichuan in 2017, we included 181 counties or county-level cities to meet the requirements of our panel data. The data analyzed in our study can be found in S2 Table. The specific features of the variables are shown in Table 1.

**Table 1:** Variables and data sources.

Variables	Research Subjects	Years	Data Resources
Licensed doctor density	181 counties	2010–2017	SHSY, SHEPSY
Registered nurse density	181 counties	2010–2017	SHSY, SHEPSY
Bed density	181 counties	2010–2017	SHSY, SHEPSY
The income of medical institutions per capita	181 counties	2010–2017	SHSY, SHEPSY
Outpatient visits per capita	181 counties	2010–2017	SHSY, SHEPSY
Inpatient visits per capita	181 counties	2010–2017	SHSY, SHEPSY
Local fiscal revenue per capita	181 counties	2010–2017	SSY
Gross domestic product per capita	181 counties	2010–2017	SSY
Average wage	181 counties	2010–2017	SSY
Total population	181 counties	2010–2017	SSY, CCSY
The proportion of the urban population	181 counties	2010–2017	SSY, CCSY

### Measurement of variables

#### Health resources

We regarded health resources as dependent variables, which were divided into health-related human resources, health-related material resources, and health-related financial resources. Traditionally, health-related human resources have been defined as the proportion of their quantity to the regional population and calculated as the total health-related human resources per population of 1000, which is widely applied across universities, governments, nonprofit organizations (the World Health Organization, World Bank and so on) and research institutes [23, 24]. In this study, we chose licensed doctors and registered nurses as representative personnel, who account for almost three-fifths of health-related human resources in Sichuan. Regarding health-related material resources, the density of beds in medical institutes has tended to be treated as a proxy indicator for health resources in the literature [25]. In the present study, the density of beds available in all medical institutions was treated as an indicator of health-related material resources defined as the ratio between the number of beds and the population [26]. In terms of health-related financial resources, in the Chinese context, health expenditures include governmental, social, and individual expenditures [27], respectively covering 28.3%, 43.0%, and 28.7% of total expenditures made in 2018 in China. The structure of expenditures was obtained from different medical institutions. Hence, due to data accessibility issues, we used the income of medical institutions as a proxy indicator of health expenditures. We also used the income of medical institutions per capita to represent health-related financial resources in the following formula:

$$\mathrm{Health}‐\mathrm{related}\;\mathrm{human}\;\mathrm{resources}=\frac{\mathrm{Number}\;\mathrm{of}\;\mathrm{health}‐\mathrm{related}\;\mathrm{human}\;\mathrm{resources}}{\mathrm{Population}}\times 1000$$ (1)

$$\mathrm{Health}‐\mathrm{related}\;\mathrm{material}\;\mathrm{resources}=\frac{\mathrm{Number}\;\mathrm{of}\;\mathrm{beds}}{\mathrm{Population}}\times 1000$$ (2)

$$\mathrm{Health}‐\mathrm{related}\;\mathrm{financial}\;\mathrm{resources}=\frac{\mathrm{Income}\;\mathrm{of}\;\mathrm{medical}\;\mathrm{instituations}}{\mathrm{Population}}\times 1000$$ (3)

#### Health care service demand

Health care services are regarded as one kind or a series of health services, namely, diagnosis, observation, treatment, and intervention services, designed to maintain or restore physical, mental or emotional well-being [28]. Such services are normally delivered by medical institutions of all kinds. Health care services can be categorized into inpatient and outpatient services depending on where procedures are performed and lengths of stay [29]. Outpatient facilities usually received patients with mild symptoms. After a complete diagnostic and auxiliary examination, outpatients receive a preliminary diagnosis. When outpatients require further treatment, they are admitted to an inpatient ward for further examination or surgery. Outpatients and inpatients thus reflect different health conditions. In this article, we use outpatient and inpatient visits per capita as indicators, which are computed based on the number of visits and the population [30]. We thus assume that more outpatient and inpatient visits per capita equate to greater health care service demand and vice versa.

#### Other variables

Socioeconomic and demographic indicators were included as control variables [31, 32]. Socioeconomic variables used include local fiscal revenue per capita, gross domestic product per capita, and average wages while the demographic variables used include the total population and the urban population proportion. Variable measurements, codes, and descriptions are shown in Table 2.

**Table 2:** Variables and data sources.

Variables type	Variable name	Measurement	Code	Description
dependent variable	Heath resources	Licensed doctor density	LDD	Number of licensed doctor divided by population and multiplied by 1000
		Registered nurse density	RND	The number of registered nurse divided by population and multiplied by 1000
		Bed density	BD	Number of the bed of all medical institutions divided by population and multiplied by 1000
		The income of medical institutions per capita	IMI	The income of medical institutions divided by the population
Independent variables	Health care services demand	Outpatient visits per capita	OV	Number of the outpatient visits divided by the population
		Inpatient visits per capita	IV	The number of inpatient visits divided by the population
Control variables	Socioeconomic conditions	Local fiscal revenue per capita	LFR	Local fiscal revenue divided by the population
		Gross domestic product per capita	GDP	Real Gross domestic product divided by the population
		Average wage	AW	Total wages of all employees divided by the number of all employees
	Demographic conditions	Total population	TP	The total population of all counties
		The proportion of the urban population	PUP	The total population divided by urban population and multiplied by 100

### Basis models

Previous studies conducted in other countries have proven the existence of spatial autocorrelations of health resources, which are also referred to as spillover effects or as effects of determinants originating within counties on other counties [17]. However, traditional regression analysis tools such as the OLS model cannot reflect spillover effects. Hence, we applied the spatial panel economic model to conduct a more precise analysis of health resources. We used three commonly used models proposed by Anselin [33]: the spatial lag panel model (SLPM) (Formula 1), spatial error panel model (SEPM) (Formula 2), and spatial Durbin panel model (SDPM) (Formula 3). The model used is expressed as follows:

$$\begin{array}{c} y_{it}=\rho W_{ij}y_{it}+X_{it}^{\prime }\beta +{\alpha +\mu_{i}+\gamma_{t}+\varepsilon }_{it}\\ i=1,2,3\ldots 181\;t=2010,2011\ldots 2017 \end{array}$$ (4)

The spatial lag model indicates whether a spatial correlation is temporal, showing whether the spatial weight matrix appears to be related to previous variables. The model explores whether each variable shows diffusion phenomena (spillover effects) for a given region, that is, whether the variables of specific units are affected by their adjacent areas. In the SLPM formula, parameter *β* is a regression coefficient defined similarly as in the nonspatial model, reflecting the influence of the independent variable on the dependent variable; *W_ij_* is the spatially weighted 181×181 matrix for the counties of Sichuan, describing the relationships between neighboring units, with n representing the number of counties. The spatial matrix is constructed based on whether regions share a border, the geographic distances between regions, and economic social and cultural exchanges between regions. We use the traditional strategy to construct our spatial matrix, a binary matrix with the rook criterion, holding that when two areas have a common boundary, the weight is defined as 1; otherwise, it is valued at 0. Spatial lag dependent variable *W_ij_* is an endogenous variable reflecting the effect of spatial distance on regional influence; the estimation of coefficient *ρ* results in the slope of the function, which reflects the influence of neighboring counties on the distribution of the health workforce; *μ_i_* and *γ_t_* represent spatial and time fixed effects, respectively.

$$\begin{array}{c} y_{it}=X_{it}^{\prime }\beta +\alpha +\mu_{i}+\gamma_{t}+\varepsilon_{it}\;\varepsilon_{it}=\lambda \sum_{j=i}^{n}W_{ij}\varepsilon_{it}+\tau_{it}\\ i=1,2,3\ldots 181\;t=2010,2011\ldots 2017 \end{array}$$ (5)

The spatial error model indicates that the spatial effect was in the error term; that is, the spatial weight matrix is situated within an undetectable error term, showing that the dependent variables of a specific unit are associated with the independent variables and the error term. In the SEPM formula, parameter *λ* measures the spatial dependence of observations includes in the sample, that is, the direction and degree of the influence of variable y in neighboring areas on variable y of the certain area; *ε* denotes the spatially autoregressive error term; and the other parameters are defined as they are in the SLPM.

$$\begin{array}{c} y_{it}=\rho W_{ij}y_{it}+X_{it}^{\prime }\beta +W_{ij}y_{it}\theta +\alpha +\mu_{i}+\gamma_{t}+\varepsilon_{it}\\ i=1,2,3\ldots 181\;t=2010,2011\ldots 2017 \end{array}$$ (6)

The SDPM was used to address the possibility of the dependent variables being explained by independent variables for a specific area and its surrounding units. In the SDPM formula, *θ* represents the influence of dependent variables for adjacent areas on the independent variables of a specific area. The other parameters are defined as they are in the SEPM and SLPM. Due to the existence of *W_ij_*, the coefficients of variables of the model do not reflect pure marginal effects. Changes in independent variables in one area not only affect the dependent variables for that area but also the dependent variables for other areas. Lesage and Pace in 2009 proposed direct, indirect, and total effects. For the spatial Durbin model, these can be rewritten as follows:

$$Y_{it}={\left(1-\rho W\right)}^{-1}(X_{it}\beta +WX_{it}\theta )+{\left(1-\rho W\right)}^{-1}\mu_{i}+{\left(1-\rho W\right)}^{-1}\gamma_{t}+{\left(1-\rho W\right)}^{-1}\varepsilon_{it}i$$ (7)

According to the demonstration given by Elhorst [34], one should first use the SDPM as the basic specification, and thus based on the SDPM, we explored the most suitable models for our panel data. We first used the submodel of the SDPM. Hence, we explored the following over two phases: which submodels are best suited to our data and whether the SDPM can be simplified into the SLPM and SEPM?.

For the first phase, we explored how, similar to the ordinary panel model, the spatial panel model may be divided into a spatial fixed-effect model and spatial random effect model. For the ordinary panel, the Hausman test was used to determine whether to establish a random effect model or fixed-effect model. Elhorst extended the use of the Hausman test to the spatial panel model [35]. The original hypothesis states that there is no correlation between dependent and independent variables, which means that we can accept the random-effects model. The fixed-effects model can also be divided into three types: an individual fixed model, a time fixed model, and a model with individual and time fixed effects, which are selected according to the sample size and time. For the second phase, we use Wald and LR tests to determine alternatives to the SDPM. If the Wald test requirements are met while those of the LR test are not, the SLPM is considered the best panel econometric model, and the SEPM test is chosen otherwise. The SDPM is considered the most suitable model if both tests produce negative results.

### Software tools

The spatial weight matrix was formed using GeoDa (Version 1.8.61, the University of Chicago, Chicago, IL, USA), and STATA 15.0 (Version 15.0, StataCorp, College Station, TX, USA) was employed to calculate the spatial panel models.

## Results

### The descriptive analysis

Table 3 reports the descriptive statistics for the sampled counties in Sichuan. Taking LDD as an example, the mean number of licensed doctors per 1000 people was 1.918 from 2010 to 2017 with a standard deviation of 1.580, indicating the dispersion of data. Compared to RND, LDD showed lower degrees of dispersion. The highest LDD value was almost 1000 times greater than the lowest, demonstrating tremendous health resource inequalities across Sichuan. The basic features of our four dependent variables and seven independent variables are shown. The specific relationships between the variables were analyzed with the different spatial panel models.

**Table 3:** Descriptive statistics of the variables.

Variable	Obs^1^	Mean	Std. Dev.^2^	Min. ^3^	Max. ^4^	Units
LDD	1448	1.918	1.580	0.334	33.406	Person/1000 population
RND	1448	1.787	1.770	0.189	14.733	Person/1000 population
BD	1448	4.768	3.060	0.850	19.990	/1000 population
IMI	1448	72665.25	155083.9	1032	2562716	Yuan/person
OV	1448	4.350	2.678	0.351	23.610	Times/person
IV	1448	0.152	0.092	0.000	0.701	Times/person
LFR	1448	0.168	0.261	0.000	6.514	Yuan/person
GDP	1448	30742.31	19925.53	4785	4785	Yuan
AW	1448	48340.29	13741.46	22569	22569	Yuan
TP	1448	498845.6	376088	25862	25862	Person
PUP	1448	33.156	21.411	4.6	100	%

^1^ Obs = Observations.

^2^ Std. Dev. = Standard deviation.

^3^ Min. = Minimum.

^4^ Max. = Maximum.

**Fig 2:**
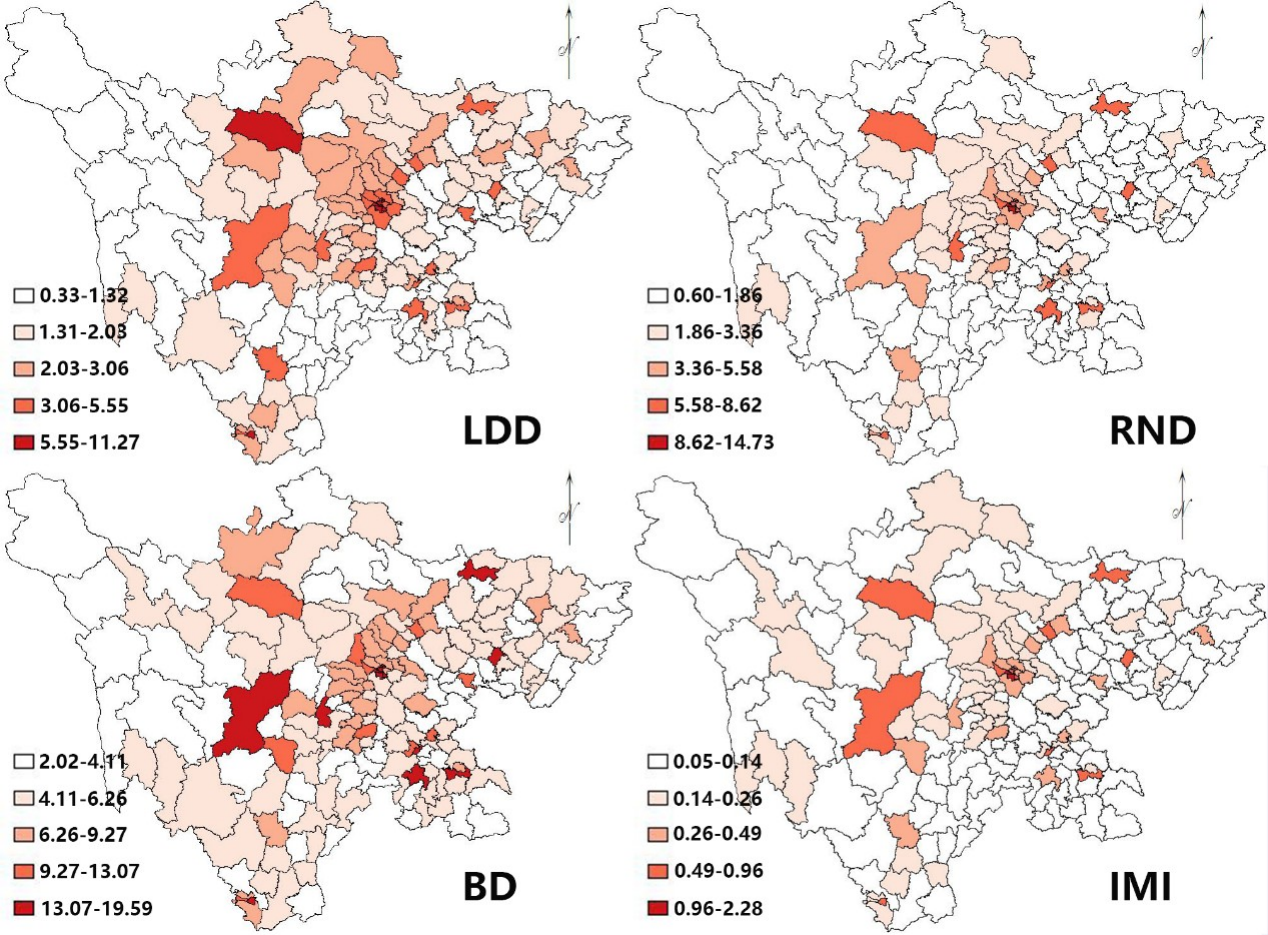
Hierarchical maps of densities of health resources. * The source of shape files was a public database, National Nature Resources and Geospatial basic information database of PRC (http://www.geodata.gov.cn/web/geo/index. html). Those shape files were under license without need for permission.

Fig 2 (the map is our own) displays hierarchical maps for different health resources for 2010 to 2017. The 181 county units were classified into five groups according to the natural breaks method based on the minimum and maximum of a set of data. Taking LDD as an example, places with most resources are located in central Sichuan and in some core geographic units in relatively poor areas. However, units located in northwestern and southeastern Sichuan show a shortage of licensed doctors. In general, the distribution of other health resources is similar to that of licensed doctors with slight differences.

### Empirical results of the spatial panel models

According to the model selection process described in the methodology section, we selected the best estimation models for different variables. The SDPM was used first. The Hausman test was applied to choose the fixed effects and random effect model. Then, the next stage was entered if we chose the random effect model, while fixed effects model was divided into the time fixed model, individual fixed model, and both fixed model according to the sample size and research period. In the context of our study, the SDPM with spatial fixed effects is used for the analysis. The second step involved using LR and Wald tests to estimate whether the SDPM could be simplified into the SLPM or SEPM, thus revealing the best model. The empirical results of all spatial panel models for health resources can be found in S3 (licensed doctors), S4 (registered nurses), S5 (beds), and S6 (income) Tables.

Table 4 displays the best estimation spatial panel models for different health resources. In this study, the SDPM was used to estimate relationships between variables in most cases, namely, LDD, BD, and IMI, and the fixed and random effects models were chosen based on the Hausman test. For RND, the SLPM with random effects was found to be the best suited model because the results failed to pass the Hausman and Wald tests.

**Table 4:** Best estimation models for estimating health resources.

Variable	LDD (SDPM with Spatial Random Effects)	RND (SLPM with Random Effects)	BD (SDPM with Spatial Fixed Effects)	IMI (SDPM with Spatial Fixed Effects)
Ln(OV)	0.152***^1^ (6.13)	0.138*** (5.37)	0.078*** (2.98)	0.082*** (3.71)
Ln(IV)	0.070*** (5.87)	0.109*** (8.13)	0.095*** (7.77)	0.067*** (6.42)
Ln(GDP)	0.324*** (7.25)	0.434*** (12.61)	0.029 (0.54)	0.085 (1.90)
Ln(AW)	-0.078 (-1.71)	0.143*** (3.83)	-0.070 (-1.46)	-0.032 (-0.78)
Ln(LFR)	-0.006 (-0.73)	0.002 (0.17)	-0.002 (0.32)	0.007 (0.92)
Ln(PUP)	0.073*** (3.57)	0.172*** (10.71)	0.056*** (2.74)	0.037* (2.14)
Ln(TP)	-0.001 (-0.15)	-0.095*** (-3.67)	-0.219* (-2.11)	0.460*** (5.23)
W × Ln(OV)	0.074 (1.75)		0.055 (1.12)	0.074 (1.76)
W × Ln(IV)	-0.081*** (-3.26)		0.066* (2.46)	0.061*** (2.70)
W × Ln(GDP)	-0.062 (-1.06)		-0.252*** (3.66)	0.480*** (7.69)
W × Ln(AW)	-0.091 (-1.56)		0.144* (2.17)	0.280*** (4.93)
W × Ln(LFR)	-0.015 (-0.89)		0.025 (1.44)	0.014 (0.97)
W × Ln(PUP)	0.030 (-1.17)		0.007 (0.28)	0.026 (1.13)
W ×Ln(TP)	-0.112* (-2.24)		0.439* (2.45)	0.550*** (3.49)
*ρ*	0.357*** (10.75)	0.261*** (6.71)	0.250*** (6.78)	0.355*** (11.59)
Log-Likelihood	637.7604	432.0649	988.4480	1223.0241
Rw^2^	0.2170	0.8134	0.7125	0.9259
Rb^2^	0.7614	0.7592	0.2241	0.7347
R^2^	0.7231	0.7645	0.2794	0.7407
Obs	1448	1448	1448	1448

^1^ *** p < 0.01

** p < 0.05

* p < 0.1.

After identifying the optimal model, a clear relationship between the independent and dependent variables was found as shown in Table 4. The results cannot be explained directly as effects of the ratio of independent variables to dependent variables due to the characteristics of spatial panel econometric models. Hence, the results are analyzed as direct, indirect (spillover), and total effects.

### Decomposition of direct, spillover and total effects

Table 5 reports direct effects of the independent variables on the different health resources. Regarding health-related human resources in Sichuan, we measure outpatient visits per capita, inpatient visits per capita, gross domestic product per capita, average wages, and the proportion of the urban population influenced the density of licensed doctors. A 1% increase in outpatient visits per capita was found to be related to a 0.163% increase in the density of licensed doctors with a 5% significance level. Similarly, an 1% increase in inpatient visits per capita, gross domestic product per capita, and the proportion of the urban population corresponded to increases of 0.065%, 0.333%, and 0.074%, respectively. For average wages, a 1% increase was associated with a 0.088% decrease in the number of licensed doctors.

**Table 5:** Direct effects of the independent variables on health resources.

Variable	LDD	RND	BD	IMI
OV	0.163***^1^ (6.47)	0.141*** (5.28)	0.082*** (3.11)	0.091*** (4.02)
IV	0.065*** (5.52)	0.110*** (8.38)	0.100*** (8.38)	0.073*** (7.17)
GDP	0.333*** (8.05)	0.443*** (13.56)	0.048 (0.98)	0.130*** (3.21)
AW	-0.088* (-2.04)	0.144*** (4.04)	-0.064 (-1.41)	-0.011 (-0.28)
LFR	-0.008 (-0.94)	0.001 (0.16)	0.004 (0.50)	0.008 (1.14)
PUP	0.074*** (3.79)	0.176*** (10.89)	0.059*** (2.94)	0.041* (2.46)
TP	-0.015 (-0.35)	-0.097*** (-3.66)	-0.198 (-1.84)	0.519*** (5.71)

^1^ *** p < 0.01

** p < 0.05

* p < 0.1.

Almost all of the dependent variables are related to the number of registered nurses except for local fiscal revenues per capita. Growth in the number of registered nurses based on a 1% increase in outpatient visits per capita, inpatient visits per capita, gross domestic product per capita, average wages, and the proportion of the urban population reaches 0.141%, 0.110%, 0.443%, 0.144%, and 0.176%, respectively, with a 5% significance level. However, a 1% increase is associated with a 0.097% increase in the number of registered nurses.

In regard to bed density, a 1% increase in outpatient visits per capita, inpatient visits per capita, and the proportion of the urban population is associated with 0.082%, 0.100%, and 0.059% increases in bed density, respectively. For the income of medical institutes, corresponding values for outpatient visits per capita, inpatient visits per capita, gross domestic product per capita, the urban population proportion and the total population are 0.091%, 0.073%, 0.130%, 0.041%, and 0.519%, respectively.

Table 6 reports the spillover effects of independent variables on different health resources of surrounding areas. Regarding outpatient visits per capita and inpatient visits per capita, the findings show that both play important roles in driving almost all health resources. It was estimated that with a 1% increase in outpatient and inpatient visits per capita in a specific geographic unit, the density of licensed doctors in adjacent counties increases and decreases by 0.195% and 0.082%, respectively, while numbers of registered nurses in surrounding areas increase by 0.047% and 0.037%, respectively, corresponding to 1% of two-way visits per capita. However, only inpatient visits per capita can influence the bed density of adjacent areas, showing a 0.118% increase corresponding to 1% of inpatient patients per capita. For medical institute income, with 1% increase in the OV and IV of one specific county, the IMI of surrounding areas increases by 0.149% and 0.128%, respectively.

**Table 6:** Spillover effects of independent variables on health resources.

Variable	LDD	RND	BD	IMI
OV	0.195***^1^ (3.32)	0.047*** (4.26)	0.093 (1.59)	0.149*** (2.64)
IV	-0.082* (-2.21)	0.037*** (5.36)	0.118*** (3.64)	0.128*** (4.04)
GDP	0.071 (1.10)	0.147*** (7.40)	0.330*** (4.17)	0.748*** (10.14)
AW	-0.170* (-2.39)	0.047*** (4.15)	0.156* (1.98)	0.390*** (5.23)
LFR	-0.03 (-1.15)	0.000 (0.15)	0.034 (1.47)	0.025 (1.13)
PUP	-0.001 (-0.20)	0.059*** (6.25)	0.027 (0.97)	0.057* (2.21)
TP	-0.170*** (-2.87)	-0.032*** (-3.24)	0.502* (2.30)	1.059*** (5.04)

^1^ *** p < 0.01

** p < 0.05

* p < 0.1.

Concerning gross domestic product per capita, average wages, and local fiscal revenues per capita we find no significant results for local fiscal revenues per capita, showing no spillover effect between LRF and different health resources. Gross domestic product per capita tends to have a greater influence than average wages for RND, BD, and IMI, which show increases of 0.147%, 0.330%, and 0.748% in surrounding areas with a 1% increase in the local unit, while the relatively low coefficients of average wages are 0.047%, 0.156%, and 0.390%, respectively. Effects on LDD show different patterns, with a 1% increase in the average wage in the specific unit related to a decrease of 0.170% in LDD in adjacent counties.

For proportions of the urban and total populations, PUP shows spillover effects on RND and IMI, and a 1% increase in PUP in the local county is associated with 0.059% and 0.057% increases in RND and IMI in adjacent counties, respectively. In regard to the total population, an increase of 1% in the local unit corresponds to 0.170% and 0.032% decreases in LDD and RND in surrounding areas, respectively, while the coefficients are 0.502% and 1.059% for BD and IMI, respectively.

### Comparison and visualization of direct and spillover effects

**Fig 3:**
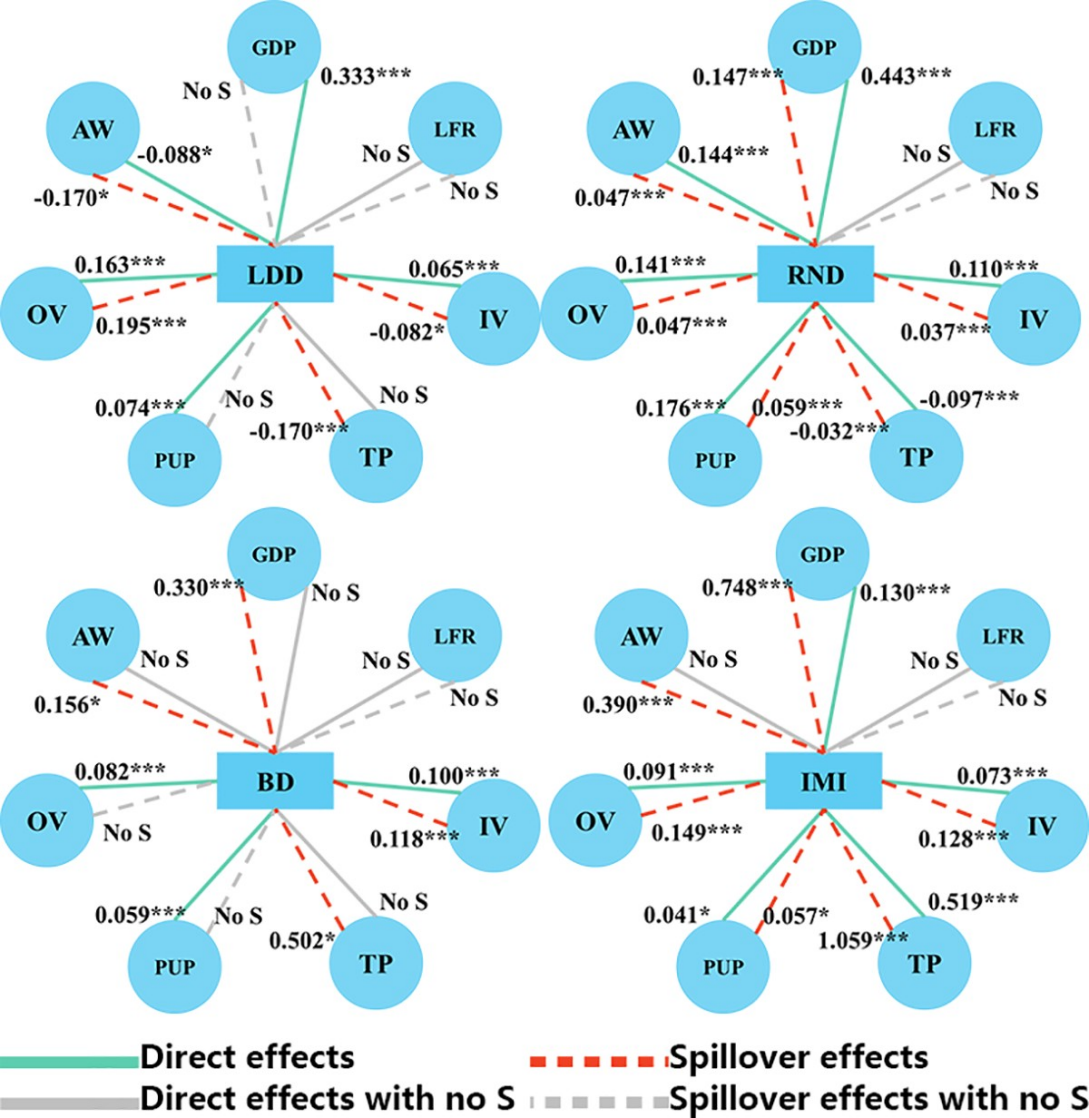
Summary of direct and spillover effects of variables on the different health resources.

Fig 3 summarizes the direct (green solid line) and spillover effects (red dashed line) of all of the variables on the different health resources. On the one hand, the regression results show significance direct effects of both outpatient and inpatient health services per capita on health resources, demonstrating that health demand is a core factor driving the density of health resources. On the other hand, the findings confirm a positive correlation between outpatient visits per capita in one local unit and licensed doctors per capita, registered nurses per capita, and medical institute income per capita in surrounding areas, while we fail to find significant relations between bed density and outpatient visits per capita. While a positive correlation was found between inpatient visits per capita for specific units and registered nurses per capita, beds per capita, and medical institute income per capita in adjacent areas, a negative correlation was found between inpatient visits in one area and LDD in the bordering region.

As shown by the red dashed line in Fig 3, a county’s growing health needs could affect the density of different health resources in surrounding areas in two ways. The negative correlations between the different variables imply that increased health needs in one county are accompanied not only by a higher density of health resources in the local region but also by a decrease in the density of health resources in surrounding areas, such as inpatient visits and licensed doctor density. In contrast, the positive correlation found means that an increase in health resources in one area could attract health resources for adjacent counties.

In addition, from a socioeconomic perspective, gross domestic product per capita and average wages play different roles in health resources. Regarding direct effects, gross domestic product per capita shows a strong relationship with different health resources except in the case of bed density, while average wages show a positive relationship to the number of registered nurses and, surprisingly, a negative relationship with licensed doctors. For indirect effects, gross domestic product per capita in one area attracts health resources to surrounding areas. However, average wages show two effects with an increase in average wages in one unit attracting licensed doctors from surrounding areas and other health resources to adjacent areas.

From a demographic perspective, regarding direct effects, the total population is negatively associated with the number of registered nurses, which proves easier to explain with the calculations. In contrast, the total population is negatively related to medical institute income. An increase in the proportion of the urban population significantly improves the number of health resources available. Regarding indirect effects, an increase in the total population of a specific region leads to a decrease in health-related human resources and an increase in other health-related resources in surrounding areas. An increase in the proportion of the urban population leads to an increase in the number of registered nurses and in medical institute income in adjacent areas.

## Discussion

This study employed county-level panel data for 181 counties in Sichuan, China for 2010 to 2017 to describe the distribution of health resources and to empirically explore the county-level determinants of densities of different health resources in Sichuan. Health care service demand, socioeconomic and demographic variables, and spillover effects drive how health resources are distributed. Above all, with the exception of local fiscal revenue per capita, almost all direct and spillover effects of the other independent variables can achieve statistical significance for at least one type of health resource, indicating the rationality and validity of the models.

In terms of health care service demand, health care services have a considerable influence on health resources at the county level in Sichuan; however, outpatient and inpatient health care service demands play different roles in driving health resources. According to the empirical results of this work, the densities of all health resources are significantly associated with health care service demand. Previous studies have paid more attention to the relationship between health care service demand and health resource distribution. Classical health economists believe that demand for health service care services is health derived [36]. According to the theory proposed by Michael Grossman [37], two factors could contribute to demand for health services: health can be treated as a consumer product or as an investment product.

According to the WHO’s definition of health service care demand [38] and the six principles defined by the American Agency for Healthcare Research and Quality [39], there are six principles of health service care demand: safety, efficiency, effectiveness and timeliness, fairness, cost effectiveness and patient orientation, which are all related to the allocation of health resources. Discussion of the relationship between health service care demand and resources has always been a contested topic [40]. Similar results have been found for Sweden [41], India [42], and Malawi [43].

Regarding spillover effects, two opposing effects were found from the empirical results of the spatial panel econometric model, namely, effects of attraction within bordering counties and effects of attraction from outside of bordering counties. However, only the densities of licensed doctors can be used to measure attraction within border counties. It is not surprising that licensed doctors are more easily drawn to high-demand areas, echoing the results given by Zhu et al. [17]. All other health resources show a positive correlation with outpatient and inpatient visits. Interestingly, health services show a stronger relationship with the bed density and income of medical institutes than health-related human resources, showing that material and financial resources are easier to relocate than human resources. Unfortunately, we failed to identify a relationship between bed density in the surrounding area and outpatient visits in a specific unit, as outpatients are not hospitalized.

The findings of this paper demonstrate that the Chinese government should pay more attention to health service care demand to achieve a more reasonable allocation of health resources. The Chinese, government currently uses the density of health resources to adjust their distribution without considering demand determinants in policy making. On the one hand, considerable variations in health service care demand can be found in Sichuan Province, demonstrating that pressures on health service care vary across the region. On the other hand, health resources cannot just be allocated based on the population of a given region, which can use health resources to configure health resources. For a long time, a dominant theory was followed in the allocation of health resources. Due to information asymmetry in health services, the excessive allocation of resources easily induces consumption, requiring the allocation of health resources to be regulated. However, at present, a relatively complete health insurance system to some extent guides medical institutions and doctors in reducing induced consumption. At the same time, with improvements in citizens’ health literacy and declines in information acquisition costs brought about by the emergence of the Internet, problems of "information asymmetry" in the health field have been effectively alleviated. Therefore, this issue should not continue to focus on health resource allocation but should be based on demand and problem orientation.

In terms of socioeconomic conditions, we found no relationship between local fiscal revenues per capita and health resources. However, Pan et al. found a relationship between fiscal revenues and health resources [18]. Local fiscal revenue was partly originates from central government transfers. The Chinese government has tended to increase investment in impoverished areas. Thus, government health expenditures are a more suitable variable for such research. A positive relationship between health resources and gross domestic product per capita is found except in the case of bed density. Li et al. found a positive relationship between bed density and gross domestic product per capita. Regarding average wages, there are no significant relationships wage and bed density and medical institute income, which is more intuitive.

Regarding spillover effects, this study find a strong relationship between gross domestic product per capita in one area and health resources in an adjacent region except in the case of the number of licensed doctors. The average wage in one county may lead to a decrease in the number of doctors in surrounding areas and to an increase in other health resources, demonstrating a coexistence of effects of attraction within and from outside of bordering counties. Economic conditions were found to be a particularly powerful factor in our model. The relationship between average wages and health resources partly reflects the influence of out-of-pocket expenses, which should be covered by other expenditures such as government and social expenditures. At the same time, the relationship found between GDP and health resources is reasonable in light of China’s health financing policies. However, this situation could lead to wealthier regions being able to afford to invest in health resources while poorer regions cannot.

The total population shows a negative relationship with the number of registered nurses and a positive relationship with medical institute income. This is evident from the empirical results. The proportion of the urban population is positively associated with all health resources. Regarding spillover effects, the total population in one region can conversely affect the number of licensed doctors and registered nurses but is positively related to bed density and medical institute income.

This study presents some limitations. First, the data used do not cover all of the counties in Sichuan due to changes in administrative division and data availability limitations. Second, there were few provincial- and municipal-level analyses to draw from. Third, our selection of independent variables was limited due to issues of data availability. While more socioeconomic factors should be considered, they were not considered in this study.

## Conclusion

For the Chinese context, this study explored the relationship between health resources and factors of health care service demand and socioeconomic and demographic perspectives and measured coefficients using spatial models and data from Sichuan Province covering eight consecutive years (2010–2017). According to our results, the spatial models tested work well in identifying the distribution of health resources from a macroscale perspective. Additionally, spillover effects were found through our analysis, showing that the studied factors could significantly influence the distribution of health resources in surrounding areas.

Our study applied a macrospatial perspective to understand health resource distribution and changing patterns of different counties in Sichuan Province and stresses the need for spatial analyses in further research. Our results provide the Chinese government with more evidence to formulate and promulgate effective policies, especially those aiming to tackle inequity between regions. On the one hand, the Sichuan government should consider health care service demand when enacting and implementing policies. An inability to satisfy patients’ needs, the movement of patients, and the loss of resources create vicious patterns. When the resources of a specific county cannot satisfy the needs of its citizens, this leads to distrust and a loss of patients. A shortage of patients then causes the flow of health resources with patients, which brings about resource losses in the specific region. In short, the more the needs of patients are satisfied, the more health resources are retained in given counties. The Sichuan government must spare no effort in protecting patients within the region. On the other hand, there is no harm in also promoting socioeconomic development, population growth, and urbanization as part of direct and effective decision-making.

## Supporting information

S1 File (PDF)

S1 Fig The administrative and topographic units of Sichuan province. (DOCX)

S1 Table Summarized data of Sichuan province. (XLSX)

S2 Table Estimation results of spatial panel econometric models for LDD. (DOCX)

S3 Table Estimation results of spatial panel econometric models for RND. (DOCX)

S4 Table Estimation results of spatial panel econometric models for BD. (DOCX)

S5 Table Estimation results of spatial panel econometric models for IMI. (DOCX)

## List of abbreviations

WHO: World Health Organization
CT: computed tomography
MRI: magnetic resonance imaging
SHSY: Sichuan health Statistics Yearbook
SHFPSY: Sichuan Health and Family Planning Statistical Yearbook
SSY: Sichuan Statistical Yearbook
CCSY: Sichuan Bureau of Statistics and China County Statistical Yearbook
LDD: Licensed doctor density
RND: Registered nurse density
BD: Bed density
IMI: Income of medical institutions per capital
OV: Outpatient visits per capita
IV: Inpatient visits per capital
LFR: Local fiscal revenue per capital
GDP: Gross domestic product per capita
AW: Average wage
TP: Total population
PUP: Proportion of urban population
OLS: Ordinary least squares
SLPM: Spatial Lag Panel Model
SEPM: Spatial Error Panel Model
SDPM: Spatial Durbin Panel Model
USA: The United States
IL: Illinois
TX: Texas
Obs: Observations
Std: Dev.: Standard deviation
Min: Minimum
Max: Maximum
