# Supplementary material for: Addressing the maldistribution of health resources in Sichuan Province, China: A county-level analysis
Source: PLoS One. 2021 Apr 23;16(4):e0250526. doi: 10.1371/journal.pone.0250526 (PMC8064550; doi:10.1371/journal.pone.0250526)
Supplement: S1 Fig — (DOCX) [file pone.0250526.s002.docx]

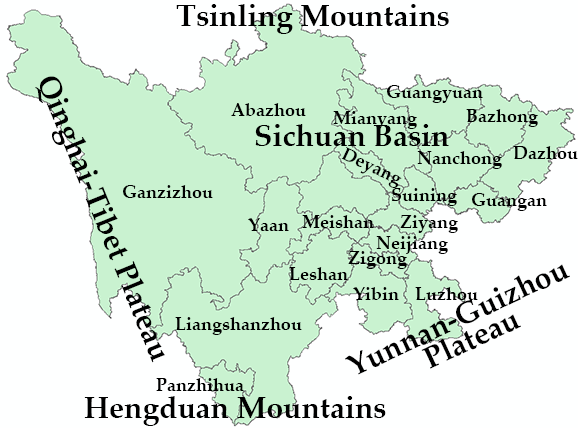


S1 Fig. The administrative and topographic units of Sichuan province

* The source of shape files was a public database, National Nature Resources and Geospatial basic information database of PRC (<http://www.geodata.gov.cn/web/geo/index>. html). Those shape files were under license without need for permission.
