# Supplementary material for: Addressing the maldistribution of health resources in Sichuan Province, China: A county-level analysis
Source: PLoS One. 2021 Apr 23;16(4):e0250526. doi: 10.1371/journal.pone.0250526 (PMC8064550; doi:10.1371/journal.pone.0250526)
Supplement: S2 Table — (DOCX) [file pone.0250526.s004.docx]

**S2 Table.** Estimation results of spatial panel econometric models for LDD.

| Variable | SDPM with individual Fixed Effects | SDPM with Time Fixed Effects | SDPM with individual and Time Fixed Effects | SDPM with Random Effects  (Best Model) | SEPM with Random  Effects | SLPM with Random  Effects |
| --- | --- | --- | --- | --- | --- | --- |
| Ln(OV) | 0.082***^1^  (3.71) | 0.294***  (15.55) | 0.055*  (2.29) | 0.152***  （6.13） | - | 0.141***  （6.06） |
| Ln(IV) | 0.067***  (6.42) | 0.198***  (13.84) | 0.038***  (3.42) | 0.070***  （5.87） | - | 0.058***  （5.07） |
| Ln(GDP) | 0.085  (1.90) | 0.237***  (9.28) | 0.048  (0.97) | 0.324***  （7.25） | - | 0.236***  （8.27） |
| Ln(AW) | -0.031  (-0.78) | 0.185***  (4.19) | -0.048  (-1.04) | -0.078  （-1.71） | - | -0.171***  （-5.38） |
| Ln(LFR) | 0.007  (0.92) | -0.028***  (-2.60) | -0.008  (-1.06) | -0.006  （-0.73） | - | -0.011  （-1.35） |
| Ln(PUP) | 0.037*  (2.14) | 0.412***  (20.90) | -0.003  (-0.16) | 0.073***  （3.57） | - | 0.044***  （3.17） |
| Ln(TP) | 0.460***  (5.23) | 0.003  (0.835) | -0.327***  (-3.43) | -0.001  （-0.15） | - | -0.087***  （-3.63） |
| W × Ln(OV) | −0.074  (1.76) | -0.089***  (-2.70) | 0.115*  (2.40) | 0.074  （1.75） |  |  |
| W × Ln(IV) | −0.061***  (2.70) | 0.025  (0.88) | -0.067***  (-2.66) | -0.081***  （-3.26） |  |  |
| W × Ln(GDP) | 0.480***  (7.69) | -0.231***  (-5.81) | 0.172  (1.68) | -0.062  （-1.06） |  |  |
| W × Ln(AW) | 0.280***  (4.93) | 0.197*  (2.52) | 0.083  (0.91) | -0.091  （-1.56） |  |  |
| W × Ln(LFR) | 0.014  (0.97) | -0.027  (-1.48) | -0.016  (-1.02) | -0.015  （-0.89） |  |  |
| W × Ln(PUP) | 0.026  (1.13) | -0.175***  (-5.03) | 0.011  (0.36) | 0.030  （-1.17） |  |  |
| W ×Ln(TP) | 0.550***  (3.49) | -0.114***  (-6.71) | -0.192  (-1.12) | -0.112*  （-2.24） |  |  |
| $\boldsymbol{\rho}$ | 0.356***  (10.90) | 0.416***  (12.81) | 0.323***  (9.54) | 0.357***  （10.75） |  | 0.350***  （11.15） |
| λ |  |  |  |  | - |  |
| LL | 1223.0241 | 145.6503 | 1119.3096 | 637.7604 | - | 624.0031 |
| Rw^2^ | 0.9259 | 0.1840 | 0.2516 | 0.2170 | - | 0.2216 |
| Rb^2^ | 0.7347 | 0.9025 | 0.0336 | 0.7614 | - | 0.7152 |
| R^2^ | 0.7407 | 0.6601 | 0.0374 | 0.7231 | - | 0.6760 |
| Obs | 1448 | 1448 | 1448 | 1448 | 1448 | 1448 |

^1^ *** p < 0.01, ** p < 0.05, * p < 0.1.
