# Supplementary material for: Addressing the maldistribution of health resources in Sichuan Province, China: A county-level analysis
Source: PLoS One. 2021 Apr 23;16(4):e0250526. doi: 10.1371/journal.pone.0250526 (PMC8064550; doi:10.1371/journal.pone.0250526)
Supplement: S3 Table — (DOCX) [file pone.0250526.s005.docx]

**S3 Table.** Estimation results of spatial panel econometric models for RND.

| Variable | SDPM with individual Fixed Effects | SDPM with Time Fixed Effects | SDPM with individual and Time Fixed Effects | SDPM with Random Effects | SEPM with Random  Effects | SLPM with Random  Effects  (Best Model) |
| --- | --- | --- | --- | --- | --- | --- |
| Ln(OV) | 0.038  (1.39) | 0.252***^1^  (10.67) | 0.029  (1.06) | 0.142***  （5.02） | 0.160***  (6.10) | 0.138***  （5.37） |
| Ln(IV) | 0.066***  (5.10) | 0.297***  (16.74) | 0.058***  (4.59) | 0.108***  （7.79） | 0.117***  (8.50) | 0.109***  （8.13） |
| Ln(GDP) | 0.077  (1.39) | 0.302***  (9.57) | 0.022  (0.39) | 0.378***  （7.25） | 0.536***  (16.03) | 0.434***  （12.61） |
| Ln(AW) | 0.093  (1.88) | 0.347***  (6.36) | -0.050  (0.95) | 0.106*  （2.02） | 0.275***  (7.58) | 0.143***  （3.83） |
| Ln(LFR) | 0.001  (0.16) | -0.013  (-1.01) | 0.001  (0.16) | 0.001  （0.12） | 0.007  (0.68) | 0.002  （0.17） |
| Ln(PUP) | 0.136***  (6.37) | 0.598***  (24.49) | 0.137***  (6.37) | 0.211***  （8.95） | 0.213***  (11.76) | 0.172***  （10.71） |
| Ln(TP) | -0.622***  (-5.80) | 0.037*  (2.19) | -0.622***  (-5.80) | -0.027  （-0.51） | -0.102***  (-4.13) | -0.095***  （-3.67） |
| W × Ln(OV) | 0.012  (0.22) | -0.181***  (-4.68) | 0.012  (0.22) | -0.018  （-0.37） |  |  |
| W × Ln(IV) | 0.057*  (2.04) | 0.054  (1.53) | -0.017  (0.62) | 0.026  （0.90） |  |  |
| W × Ln(GDP) | 0.413***  (5.61) | -0.192***  (-5.81) | 0.015  (0.13) | 0.121  （1.69） |  |  |
| W × Ln(AW) | 0.040  (0.57) | 0.289***  (2.97) | -0.114  (-1.10) | 0.031  （0.46） |  |  |
| W × Ln(LFR) | -0.013  (-0.70) | -0.001  (-0.04) | -0.008  (-0.41) | -0.015  （-0.77） |  |  |
| W × Ln(PUP) | 0.024  (0.85) | -0.156***  (-3.53) | 0.111***  (3.17) | -0.051  (-1.70) |  |  |
| W ×Ln(TP) | 0.435*  (2.31) | -0.151***  (-7.31) | 0.182  (0.94) | -0.103  (-1.69) |  |  |
| $\boldsymbol{\rho}$ | 0.268***  (7.58) | 0.211***  (5.65) | 0.143***  (9.54) | 0.233***  （6.40） |  | 0.261***  （6.71） |
| λ |  |  |  |  | 0.243***  (6.20) |  |
| LL | 920.3849 | -142.6637 | 959.8945 | 437.6220 | 415.8569 | 432.0649 |
| Rw^2^ | 0.8239 | 0.7645 | 0.6162 | 0.8150 | 0.8055 | 0.8134 |
| Rb^2^ | 0.4353 | 0.9018 | 0.0568 | 0.7724 | 0.7932 | 0.7592 |
| R^2^ | 0.5024 | 0.8240 | 0.0792 | 0.7745 | 0.7913 | 0.7645 |
| Obs | 1448 | 1448 | 1448 | 1448 | 1448 | 1448 |

^1^ *** p < 0.01, ** p < 0.05, * p < 0.1.
