# Supplementary material for: Addressing the maldistribution of health resources in Sichuan Province, China: A county-level analysis
Source: PLoS One. 2021 Apr 23;16(4):e0250526. doi: 10.1371/journal.pone.0250526 (PMC8064550; doi:10.1371/journal.pone.0250526)
Supplement: S4 Table — (DOCX) [file pone.0250526.s006.docx]

**S4 Table.** Estimation results of spatial panel econometric models for BD.

| Variable | SDPM with individual Fixed Effects  (Best Model) | SDPM with Time Fixed Effects | SDPM with individual and Time Fixed Effects | SDPM with Random Effects | SEPM with individual Fixed Effects | SLPM with individual Fixed Effects |
| --- | --- | --- | --- | --- | --- | --- |
| Ln(OV) | 0.078***^1^  （2.98） | 0.200***  (9.94) | 0.077***  (2.98) | 0.169***  (6.70) | 0.128***  (4.88) | 0.107***  (4.27) |
| Ln(IV) | 0.095***  （7.77） | 0.333***  (21.90) | 0.090***  (7.33) | 0.139***  (10.44) | 0.120***  (9.62) | 0.112***  (9.32) |
| Ln(GDP) | 0.029  （0.54） | 0.189***  (6.98) | -0.007  (-0.13) | 0.276***  (6.42) | 0.379***  (10.58) | 0.260***  (7.71) |
| Ln(AW) | -0.070  （-1.46） | 0.137***  (2.91) | -0.074  (-1.46) | -0.041  (-0.84) | 0.174***  (4.38) | 0.077*  (2.05) |
| Ln(LFR) | -0.002  （0.32） | -0.030***  (-2.66) | 0.006  (0.67) | 0.001  (0.07) | 0.009  (0.99) | 0.006  (0.74) |
| Ln(PUP) | 0.056***  （2.74） | 0.330***  (15.75) | 0.065***  (3.12) | 0.129***  (5.98) | 0.090***  (5.40) | 0.066***  (4.50) |
| Ln(TP) | -0.219*  （-2.11） | 0.057***  (3.95) | -0.250*  (-2.41) | 0.069  (1.77) | -0.001  (-0.01) | -0.050  (-0.53) |
| W × Ln(OV) | 0.055  （1.12） | -0.041  (-1.22) | 0.044  (0.86) | -0.023  (-0.52) |  |  |
| W × Ln(IV) | 0.066*  （2.46） | -0.027  (-0.88) | 0.039  (1.43) | 0.025  (0.90) |  |  |
| W × Ln(GDP) | -0.252***  （3.66） | -0.194***  (-4.59) | 0.012  (0.11) | -0.027  (-0.46) |  |  |
| W × Ln(AW) | 0.144*  （2.17） | 0.305***  (3.70) | 0.117  (1.16) | 0.184***  (3.01) |  |  |
| W × Ln(LFR) | 0.025  （1.44） | -0.026  (-1.34) | 0.037*  (2.08) | 0.011  (0.64) |  |  |
| W × Ln(PUP) | 0.007  （0.28） | -0.056  (-1.55) | 0.068*  (2.03) | -0.073***  (-2.69) |  |  |
| W ×Ln(TP) | 0.439*  （2.45） | -0.164***  (-9.20) | 0.334  (1.81) | -0.156***  (-3.46) |  |  |
| $\boldsymbol{\rho}$ | 0.250***  （6.78） | -0.181***  (4.53) | 0.193***  (5.22) | 0.241***  (6.65) |  | 0.340***  (10.74) |
| λ |  |  |  |  | 0.263***  (7.09) |  |
| LL | 988.4480 | 80.5570 | 1008.6892 | 559.5265 | 935.2224 | 963.4533 |
| Rw^2^ | 0.7125 | 0.6262 | 0.6623 | 0.6993 | 0.6888 | 0.7020 |
| Rb^2^ | 0.2241 | 0.8638 | 0.3121 | 0.7435 | 0.6690 | 0.6454 |
| R^2^ | 0.2794 | 0.7788 | 0.3632 | 0.7255 | 0.6727 | 0.6554 |
| Obs | 1448 | 1448 | 1448 | 1448 | 1448 | 1448 |

^1^ *** p < 0.01, ** p < 0.05, * p < 0.1.
