# Supplementary material for: Addressing the maldistribution of health resources in Sichuan Province, China: A county-level analysis
Source: PLoS One. 2021 Apr 23;16(4):e0250526. doi: 10.1371/journal.pone.0250526 (PMC8064550; doi:10.1371/journal.pone.0250526)
Supplement: S5 Table — (DOCX) [file pone.0250526.s007.docx]

**S5 Table.** Estimation results of spatial panel econometric models for IMI.

| Variable | SDPM with individual Fixed Effects  (Best Model) | SDPM with Time Fixed Effects | SDPM with individual and Time Fixed Effects | SDPM with Random Effects | SEPM with individual Fixed Effects | SLPM with individual Fixed Effects |
| --- | --- | --- | --- | --- | --- | --- |
| Ln(OV) | 0.082***^1^  （3.71） | 0.422***  (16.76) | 0.075***  (3.47) | 0.142***  (5.99) | 0.171***  (6.72) | 0.106***  (4.93) |
| Ln(IV) | 0.067***  （6.42） | 0.343***  (18.00) | 0.601***  (5.95) | 0.090***  (7.98) | 0.113***  (9.32) | 0.082***  (7.93) |
| Ln(GDP) | 0.085  （1.90） | 0.152***  (4.48) | 0.005  (0.11) | 0.225***  (4.85) | 0.801***  (22.24) | 0.368***  (10.96) |
| Ln(AW) | -0.032  （-0.78） | 0.682***  (11.55) | -0.031  (-0.72) | 0.001  (0.03) | 0.471***  (12.13) | 0.155***  (4.53) |
| Ln(LFR) | 0.007  （0.92） | -0.043***  (-3.04) | 0.010  (1.34) | 0.007  (0.91) | 0.017*  (2.04) | 0.010  (1.44) |
| Ln(PUP) | 0.037*  （2.14） | 0.533***  (20.27) | 0.038***  (2.22) | 0.081***  (4.26) | 0.108***  (6.52) | 0.056***  (4.46) |
| Ln(TP) | 0.460***  （5.23） | 1.083***  (60.43) | 0.396***  (4.56) | 0.867***  (13.35) | 0.909***  (9.01) | 0.607***  (7.33) |
| W × Ln(OV) | 0.074  （1.76） | -0.213***  (-4.94) | 0.029  (0.67) | 0.0232  (0.54) |  |  |
| W × Ln(IV) | 0.061***  （2.70） | -0.107***  (-2.87) | 0.037  (1.61) | 0.023  (0.97) |  |  |
| W × Ln(GDP) | 0.480***  （7.69） | -0.250***  (-4.73) | -0.030  (-0.33) | 0.308***  (4.70) |  |  |
| W × Ln(AW) | 0.280***  （4.93） | 0.596***  (5.40) | 0.256***  (3.03) | 0.339***  (5.72) |  |  |
| W × Ln(LFR) | 0.014  （0.97） | 0.036  (1.49) | 0.023  (1.54) | 0.008  (0.51) |  |  |
| W × Ln(PUP) | 0.026  （1.13） | -0.181***  (-3.92) | 0.048  (1.72) | -0.041  (-1.66) |  |  |
| W ×Ln(TP) | 0.550***  （3.49） | -0.427***  (-10.36) | 0.390*  (2.47) | -0.306***  (-3.79) |  |  |
| $\boldsymbol{\rho}$ | 0.355***  （11.59） | 0.288***  (8.07) | 0.227***  (6.19) | 0.347***  (10.49) |  | 0.564***  (23.30) |
| λ |  |  |  |  | 0.307***  (7.38) |  |
| LL | 1223.0241 | -254.0961 | 1263.5113 | 648.6570 | 981.9690 | 1155.0452 |
| Rw^2^ | 0.9259 | 0.8268 | 0.8692 | 0.9220 | 0.8977 | 0.9213 |
| Rb^2^ | 0.7347 | 0.9622 | 0.7296 | 0.8519 | 0.8859 | 0.7923 |
| R^2^ | 0.7407 | 0.9168 | 0.7155 | 0.8583 | 0.8865 | 0.7964 |
| Obs | 1448 | 1448 | 1448 | 1448 | 1448 | 1448 |

^1^ *** p < 0.01, ** p < 0.05, * p < 0.1.
